# Impact of left anterior descending lesion location on midterm outcomes in patients undergoing left internal mammary artery grafting: a five-year cohort study integrating quantitative flow ratio assessment

**DOI:** 10.3389/fcvm.2025.1605573

**Published:** 2025-08-11

**Authors:** Ziang Sun, Yuhui Wu, Rui Jiang, Zeyu Chen, Tianyu Wang, Wenlong Yan, Sumin Yang

**Affiliations:** ^1^ Department of Cardiovascular Surgery, The Affiliated Hospital of Qingdao University, Qingdao University, Qingdao, China; ^2^ Department of DSA Room, The Affiliated Hospital of Qingdao University, Qingdao, China; ^3^ School of Medicine and Pharmacy, Ocean University of China, Qingdao, China

**Keywords:** quantitative flow ratio (QFR), coronary artery bypass grafting (CABG), left anterior descending artery (LAD), lesion localization, angiography

## Abstract

**Background:**

The prognostic value of coronary artery bypass grafting (CABG) may be suboptimal when guided solely by anatomical stenosis severity. Quantitative flow ratio (QFR), a computational angiography-derived hemodynamic assessment tool, offers functional insights; however, its prognostic interplay with lesion localization [proximal vs. mid-to-distal left anterior descending artery (LAD)] remains unclear. This study evaluates the impact of QFR-guided revascularization, stratified by LAD lesion location, on midterm clinical outcomes.

**Methods:**

A retrospective cohort of 481 patients undergoing left internal mammary artery (LIMA) to LAD grafting (2019–2023) was analyzed. Lesions were classified as proximal (Site 1) or mid-to-distal (Site 2) LAD and stratified by QFR thresholds (High: ≥0.80; Low: <0.80). The primary endpoint was 5-year major adverse cardiovascular and cerebrovascular events (MACCEs), assessed using Kaplan–Meier survival analysis and Cox regression.

**Results:**

High QFR patients (*n* = 139) exhibited lower diabetes (28.1% vs. 40.6%, *p* = 0.013), smoking rates (27.3% vs. 38.6%, *p* = 0.025), and 3-vessel disease (48.9% vs. 74.6%, *p* < 0.0001) compared to low QFR (*n* = 342). Proximal lesions with high QFR had markedly higher MACCEs risk (HR = 1.91, 95% CI: 1.18–3.10; *Log-rank P* = 0.0075), whereas mid-to-distal lesions showed no QFR-driven prognostic differences (*p* = 0.46). Lesion location alone did not independently influence survival (*Log-rank P* = 0.8).

**Conclusion:**

QFR-guided risk stratification is most prognostically impactful for proximal LAD lesions, where hemodynamic significance plays a critical role in clinical outcomes. In contrast, mid-to-distal lesions exhibit limited QFR utility, emphasizing anatomical-functional synergy in CABG planning. Despite comparable survival across lesion sites, proximal low QFR lesions warrant intensified surveillance.

## 1 Introduction

The use of left internal mammary artery (LIMA) in left anterior descending artery (LAD) bypass grafting is recognized as the gold standard in coronary artery bypass grafting (CABG) due to its superior long-term patency and well-documented survival benefits (1). However, the implantation of bypass conduits onto target vessels without significant stenosis may result in competitive flow between the native and grafted conduits, potentially compromising long-term graft patency by altering endothelial shear stress (2, 3). Furthermore, conventional coronary angiography solely identifies anatomical stenotic lesions but do not effectively clarify the physiological significance of such obstructions on myocardial perfusion territories. Consequently, relying solely on angiographic assessments may prove insufficient for optimal CABG decision-making.

Although the 2018 ESC/EACTS Guidelines on Myocardial Revascularization strongly advocate (Class I recommendation) for pressure wire-based physiological assessment, such as fractional flow reserve (FFR), for determining the hemodynamic significance of intermediate-grade stenoses (4), its clinical adoption remains limited. This is primarily attributed to procedural time constraints (typically >20 minutes per vessel), the risk of pressure wire-related complications (0.8%–2.1% incidence), the prevalence of adenosine-induced adverse effects (15%–30% prevalence), and the associated incremental healthcare costs (5, 6).

Quantitative flow ratio (QFR), an emerging angiography-derived index, utilizes three-dimensional (3D) coronary reconstruction—based on invasive angiograms—coupled with computational fluid dynamics (CFD) algorithms to compute virtual FFR values without requiring hyperemic agents or intracoronary pressure measurement. Multicenter validation trials (FAVOR II & III) (7) have demonstrated excellent diagnostic concordance between QFR and invasive FFR (89%–93% agreement rates), with the area under the curve (AUC) values exceeding 0.90 for detecting functionally significant stenoses. Additionally, QFR reduces procedural time by 7–12 minutes per case and eliminates the risks associated with pharmacological agents or pressure wire application.

Although accumulating evidence from multicenter registries supports the prognostic benefits of QFR-guided revascularization strategies in reducing the incidence of midterm major adverse cardiac events (MACE) (8–10), its applications in CABG surgical planning remain underexplored. Specifically, it remains unclear whether QFR-guided CABG significantly influences prognostic outcomes and long-term survival based on coronary lesion anatomic localization.

This study aims to evaluate the predictive value of QFR in midterm outcomes of LIMA grafts after CABG, focusing on stratification by lesion localization (proximal, mid, or distal segments) within the LAD.

## 2 Methods

### 2.1 Study design

**Figure 1:**
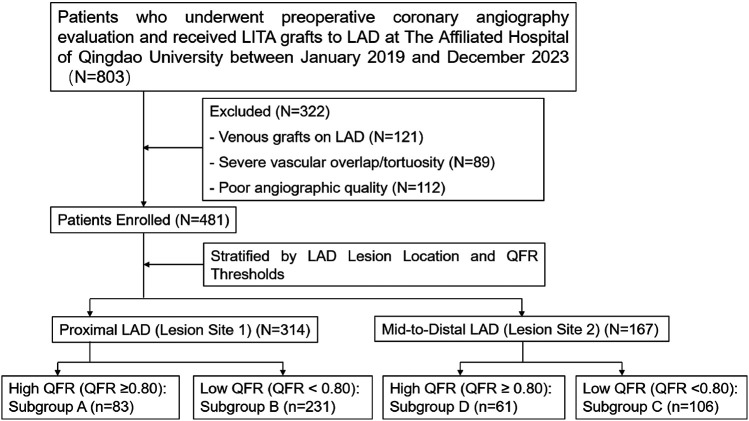
Enrollment diagram.

This study used a retrospective analysis of adult patients who underwent preoperative coronary angiography evaluation and subsequent LIMA grafts to LAD at the Affiliated Hospital of Qingdao University between January 2019 and December 2023 (Figure 1). Outcomes were tracked via institutional Electronic Medical Records (EMR) for hospitalized patients and telephone interviews for others.

**Inclusion criteria:** (1) Patients who underwent coronary angiography at our center within 6 months before surgery; (2) Presence of LAD stenosis with severity ranging from 75%–90%.

**Exclusion criteria:** (1) Patients with venous grafts on the LAD artery; (2) Severe vascular overlap or tortuosity in the stenotic segment; (3) Poor angiographic image quality precluding accurate lumen contour detection.

Coronary lesion localization was classified according to the Society of Cardiovascular Computed Tomography (SCCT) guidelines (11), grouping serial stenoses based on the originating segment of the most severe stenosis (≥70%) and stratifying LAD lesions into two anatomical subgroups:

• **Lesion Site 1**: Proximal LAD, defined as the segment extending from the ostium to the origin of the first major septal perforator.
• **Lesion Site 2**: Mid-to-distal LAD, comprising the segment distal to the first major septal perforator, including diagonal branch bifurcations.

Functional assessments were conducted using QFR, categorizing lesions into two hemodynamic groups:

• **High QFR Group**: QFR > 0.80 (indicative of functionally nonsignificant stenosis)
• **Low QFR Group**: QFR ≤ 0.80 (indicative of functionally significant stenosis)

A composite stratification was then performed by integrating anatomical and functional parameters, yielding four distinct clinical subgroups:

• **Subgroup A**: High QFR at Lesion Site 1(QFR > 0.80 with proximal LAD lesions)
• **Subgroup B**: High QFR at Lesion Site 2(QFR > 0.80 with mid-to-distal LAD lesions)
• **Subgroup C**: Low QFR at Lesion Site 1(QFR ≤ 0.80 with proximal LAD lesions)
• **Subgroup D**: Low QFR at Lesion Site 2(QFR ≤ 0.80 with mid-to-distal LAD lesions)

### 2.2 Coronary angiography

Coronary angiography was performed via radial or femoral access using 5-F/6-F sheaths. Angiographic imaging was acquired using Philips Allura Xper FD20 or GE Innova IGS520 systems at ≥15 frames/s. Intravenous unfractionated heparin (100 IU/kg) was administered before the procedure to prevent thrombotic complications.

### 2.3 QFR analysis

Lesions with a reference vessel diameter ≥1.5 mm were included in the QFR analysis. Two angiographic projections with angular separation >25° were processed using AngioPlus software version 2.1.3.0 (Pulse Medical Imaging Technology, Shanghai, China) for 3D QFR computation. Vessel contours were initially traced semi-automatically, with manual correction conducted if required. Key QFR parameters included minimal lumen diameter, area stenosis. All offline analyses were performed by two independent observers blinded to the clinical data, with discordant cases adjudicated by a third reviewer.

### 2.4 Coronary artery bypass surgery

All participants underwent coronary revascularization with the LIMA as the graft conduit for the LAD artery. The surgical approach, either off-pump or on-pump technique, was determined at the discretion of the operating surgeon based on patient-specific clinical characteristics.

### 2.5 Study end point

The primary endpoint was the cumulative incidence of 5-year MACCEs. The MACCEs were defined as a composite of all-cause mortality, cardiovascular mortality, myocardial infarction, stroke, repeat revascularization, or rehospitalization for angina pectoris during the 5-year follow-up period.

### 2.6 Statistical analysis

All statistical analyses were performed using R software (version 4.4.1). Continuous variables were expressed as mean ± standard deviation, while categorical variables were presented as frequencies (percentages). Group comparisons for continuous variables were assessed using Student's *t*-test, while Pearson's chi-squared test was used for categorical variables. Cumulative rates of MACCEs were estimated using Kaplan–Meier survival analysis, with comparisons between groups evaluated using the log-rank test. A two-tailed *p*-value < 0.05 was considered statistically significant.

## 3 Results

### 3.1 Baseline characteristics

The baseline demographic, clinical, and procedural characteristics of the study population, stratified according to the QFR thresholds and lesion locations, are summarized in Tables 1–5. The key findings are as follows:

**Table 1:** Comparison of baseline characteristics between high QFR and low QFR groups (all lesion sites combined).

Characteristic	High QFR	Low QFR	*p* value
	(*n* = 144)	(*n* = 337)	
Age (years)	63.8 ± 0.6	63.0 ± 0.4	0.275
Male	106 (76.3%)	254 (74.3%)	0.734
BMI (kg/m²)	25.4 ± 0.2	26.4 ± 0.9	0.313
Hypertension	78 (56.1%)	222 (64.9%)	0.089
Hypercholesterolemia	2 (1.4%)	10 (2.9%)	0.533
Diabetes mellitus	39 (28.1%)	139 (40.6%)	0.013
Prior MI	12 (8.6%)	50 (14.6%)	0.104
Smoking	38 (27.3%)	132 (38.6%)	0.025
Cerebrovascular diseases	17 (12.2%)	47 (13.7%)	0.768
Prior left coronary artery PCI	11 (7.9%)	15 (4.4%)	0.184
Preoperative LVEF,%	57.5 ± 0.6	55.7 ± 0.5	0.025
Concomitant surgery	49 (35.3%)	51 (14.9%)	<0.001
Valvular surgery	37 (26.6%)	36 (10.5%)	<0.001
Aortic surgery	1 (0.7%)	1 (0.3%)	>0.999
Others	6 (4.3%)	6 (1.8%)	0.190
On-pump	42 (30.2%)	33 (9.6%)	<0.001
3-vessel disease	68 (48.9%)	255 (74.6%)	<0.001
Hospital complications	76 (54.7%)	186 (54.4%)	>0.999
Hospital stays	27.6 ± 1.0	29.5 ± 0.8	0.110
NYHA class			0.962
I	3 (2.2%)	6 (1.8%)	
II	102 (73.3%)	246 (71.9%)	
III	27 (19.4%)	73(21.3%)	
IV	7(5.0%)	17(5.0%)	

BMI indicates body mass index; LVEF, left ventricular ejection fraction; MI, myocardial infarction; NYHA, New York heart association; PCI, percutaneous coronary intervention. Other concomitant surgeries included left atrial appendectomy, ventricular aneurysmectomy, and arteriovenous fistula surgery. Hospital complications included infection, bleeding, heart failure, and reoperation.

**Table 2:** Comparison of baseline characteristics between lesion site 1 and lesion site 2 (all QFR groups combined).

Characteristic	Lesion Site 1	Lesion Site 2	*p* value
	(*n* = 314)	(*n* = 167)	
Age (years)	63.1 ± 0.4	63.5 ± 0.6	0.613
Male	240 (76.4%)	120 (71.9%)	0.322
BMI (kg/m²)	25.6 ± 0.2	27.1 ± 1.8	0.395
Hypertension	203 (64.6%)	97 (58.1%)	0.188
Hypercholesterolemia	8 (2.5%)	4 (3.4%)	>0.999
Diabetes mellitus	120 (38.2%)	58 (34.7%)	0.513
Prior MI	40 (12.7%)	22 (13.1%)	>0.999
Smoking	116 (36.9%)	54 (32.3%)	0.365
Cerebrovascular diseases	43 (13.7%)	21 (12.6%)	0.839
Prior left coronary artery PCI	18 (5.7%)	8 (4.8%)	0.823
Preoperative LVEF,%	56.3 ± 0.5	56.2 ± 0.6	0.892
Concomitant surgery	50 (15.9%)	50 (29.9%)	<0.001
Valvular surgery	33 (10.5%)	40 (24.0%)	<0.001
Aortic surgery	1 (0.3%)	1 (0.6%)	>0.999
Others	8 (2.5%)	4 (2.4%)	>0.999
On-pump	32 (10.2%)	43 (25.7%)	<0.001
3-vessel disease	222 (70.7%)	101 (60.5%)	0.030
Hospital complications	161 (51.3%)	101 (60.5%)	0.067
Hospital stays	28.3 ± 0.7	30.1 ± 1.2	0.212
NYHA class			0.190
I	6 (1.9%)	3 (1.8%)	
II	237 (75.5%)	111 (66.5%)	
III	57 (18.2%)	43(25.7%)	
IV	14(4.5%)	10(6.0%)	

BMI indicates body mass index; LVEF, left ventricular ejection fraction; MI, myocardial infarction; NYHA, New York heart association; PCI, percutaneous coronary intervention. Other concomitant surgeries included left atrial appendectomy, ventricular aneurysmectomy, and arteriovenous fistula surgery. Hospital complications included infection, bleeding, heart failure, and reoperation.

**Table 3:** Comparison of baseline characteristics between high QFR and low QFR in lesion site 1 (proximal LAD).

Characteristic	Lesion Site 1-High QFR	Lesion Site 1-Low QFR	*p* value
	(*n* = 83)	(*n* = 231)	
Age (years)	63.1 ± 0.8	63.1 ± 0.5	0.946
Male	62 (79.5%)	178 (75.4%)	0.562
BMI (kg/m²)	25.5 ± 0.3	25.6 ± 0.2	0.834
Hypertension	47 (60.3%)	156 (66.1%)	0.424
Hypercholesterolemia	2 (2.6%)	6 (2.5%)	0.748
Diabetes mellitus	26 (33.3)	94 (39.8)	0.374
Prior MI	8 (10.3%)	32 (13.6%)	0.574
Smoking	23 (29.5%)	93 (39.4%)	0.150
Cerebrovascular diseases	12 (15.4%)	31 (13.3%)	0.756
Prior left coronary artery PCI	7 (9.0%)	11 (4.7%)	0.254
Preoperative LVEF,%	57.8 ± 0.8	55.8 ± 0.6	0.040
Concomitant surgery	18 (23.1%)	32 (13.6%)	0.070
Valvular surgery	12 (15.4%)	21 (8.9%)	0.160
Aortic surgery	0 (0%)	1 (0.4%)	
Others	4 (5.1%)	4 (1.7%)	0.210
On-pump	13 (16.7%)	19 (8.1%)	0.049
3-vessel disease	46 (60.0%)	176 (74.6%)	0.013
Hospital complications	39 (50.0%)	122 (51.7%)	0.897
Hospital stays	26.2 ± 1.0	29.1 ± 0.8	0.835
NYHA class			0.278
I	2 (2.6%)	4 (1.7%)	
II	61 (78.2%)	176 (74.6%)	
III	12 (15.4%)	45(19.1%)	
IV	3(3.8%)	11(4.7%)	

BMI indicates body mass index; LVEF, left ventricular ejection fraction; MI, myocardial infarction; NYHA, New York Heart Association; PCI, percutaneous coronary intervention. Other concomitant surgeries included left atrial appendectomy, ventricular aneurysmectomy, and arteriovenous fistula surgery. Hospital complications included infection, bleeding, heart failure, and reoperation.

**Table 4:** Comparison of baseline characteristics between high QFR in lesion site 1 (proximal LAD) and low QFR in lesion site 2 (Mid-to-distal LAD).

Characteristic	Lesion Site 1-High QFR	Lesion Site 2-Low QFR	*p* value
	(*n* = 83)	(*n* = 106)	
Age (years)	63.1 ± 0.8	62.7 ± 0.8	0.778
Male	62 (79.5%)	76 (71.7%)	0.301
BMI (kg/m²)	25.5 ± 0.3	28.1 ± 2.9	0.361
Hypertension	47 (60.3%)	66 (62.3%)	0.901
Hypercholesterolemia	2 (2.6%)	4 (3.8%)	0.970
Diabetes mellitus	26 (33.3)	45 (42.5%)	0.270
Prior MI	8 (10.3%)	18 (17.0%)	0.280
Smoking	23 (29.5%)	39 (36.8%)	0.380
Cerebrovascular diseases	12 (15.4%)	16 (15.1%)	>0.999
Prior left coronary artery PCI	7 (9.0%)	4 (3.8%)	0.248
Preoperative LVEF,%	57.8 ± 0.8	55.7 ± 0.8	0.066
Concomitant surgery	18 (23.1%)	19 (17.9%)	0.499
Valvular surgery	12 (15.4%)	15 (14.2%)	0.982
Aortic surgery	0 (0%)	0 (0%)	
Others	4 (5.1%)	2 (1.9%)	0.422
On-pump	13 (16.7%)	14 (13.2%)	0.657
3-vessel disease	46 (60.0%)	79 (74.5%)	0.038
Hospital complications	39 (50.0%)	64 (60.4%)	0.210
Hospital stays	26.2 ± 1.0	30.5 ± 1.5	0.021
NYHA class			0.279
I	2 (2.6%)	2 (1.9%)	
II	61 (78.2%)	70 (66.0%)	
III	12 (15.4%)	28(26.4%)	
IV	3(3.8%)	6(5.7%)	

BMI indicates body mass index; LVEF, left ventricular ejection fraction; MI, myocardial infarction; NYHA, New York heart association; PCI, percutaneous coronary intervention. Other concomitant surgeries included left atrial appendectomy, ventricular aneurysmectomy, and arteriovenous fistula surgery. Hospital complications included infection, bleeding, heart failure, and reoperation.

**Table 5:** Comparison of baseline characteristics between high QFR and low QFR in lesion site 2 (Mid-to-distal LAD).

Characteristic	Lesion Site 2-High QFR	Lesion Site 2-Low QFR	*p* value
	(*n* = 61)	(*n* = 106)	
Age (years)	64.8 ± 0.9	62.7 ± 0.8	0.088
Male	44 (72.1%)	76 (71.7%)	>0.999
BMI (kg/m²)	25.3 ± 0.4	28.1 ± 2.9	0.336
Hypertension	31 (50.8%)	66 (62.3%)	0.200
Hypercholesterolemia	34 (55.7%)	4 (3.8%)	0.321
Diabetes mellitus	13 (21.3%)	45 (42.5%)	0.009
Prior MI	4 (6.6%)	18 (17.0%)	0.093
Smoking	15 (24.6%)	39 (36.8%)	0.147
Cerebrovascular diseases	5 (8.2%)	16 (15.1%)	0.293
Prior left coronary artery PCI	4 (6.6%)	4 (3.8%)	0.664
Preoperative LVEF,%	57.0 ± 0.9	55.7 ± 0.8	0.279
Concomitant surgery	31 (50.8%)	19 (17.9%)	<0.001
Valvular surgery	25 (41.0%)	15 (14.2%)	<0.001
Aortic surgery	1 (1.6%)	0 (0%)	
Others	2 (3.3%)	2 (1.9%)	0.967
On-pump	29 (47.5%)	14 (13.2%)	<0.001
3-vessel disease	22 (36.1%)	79 (74.5%)	<0.001
Hospital complications	37 (60.1%)	64 (60.4%)	>0.999
Hospital stays	29.3 ± 1.8	30.5 ± 1.5	0.634
NYHA class			0.990
I	1 (1.6%)	2 (1.9%)	
II	41 (67.2%)	70 (66.0%)	
III	15 (24.6%)	28(26.4%)	
IV	4(6.6%)	6(5.7%)	

BMI indicates body mass index; LVEF, left ventricular ejection fraction; MI, myocardial infarction; NYHA, New York heart association; PCI, percutaneous coronary intervention. Other concomitant surgeries included left atrial appendectomy, ventricular aneurysmectomy, and arteriovenous fistula surgery. Hospital complications included infection, bleeding, heart failure, and reoperation.

#### 3.1.1 High QFR vs. low QFR

Patients with high QFR (*n* = 139) demonstrated significantly lower rates of diabetes mellitus (28.1% vs. 40.6%, *p* = 0.013), smoking (27.3% vs. 38.6%, *p* = 0.025), and 3-vessel disease (48.9% vs. 74.6%, *p* < 0.0001) compared to low QFR patients (*n* = 342). Preoperative left ventricular ejection fraction (LVEF) was higher in the high QFR group (57.5% ± 0.6% vs. 55.7% ± 0.5%, *p* = 0.025) compared to the low QFR group. High QFR patients also underwent more concomitant surgeries (35.3% vs. 14.9%, *p* < 0.0001), valvular procedures (26.6% vs. 10.5%, *p* < 0.0001), and on-pump CABG (30.2% vs. 9.6%, *p* < 0.0001) (Table 1).

#### 3.1.2 Lesion site 1 vs. lesion site 2

Lesion Site 2 was associated with greater procedural complexity, including elevated rates of concomitant surgery (29.9% vs. 15.9%, *p* = 0.0005), valvular surgery (24.0% vs. 10.5%, *p* = 0.0002), and on-pump utilization (25.7% vs. 10.2%, *p* < 0.0001). Conversely, 3-vessel disease was more prevalent in Site 1 (70.7% vs. 60.5%, *p* = 0.03). Baseline demographic characteristics and comorbidities were comparable between lesion sites (*p* > 0.05) (Table 2).

#### 3.1.3 High vs. low QFR at lesion site 1

Patients with high QFR at Lesion Site 1 exhibited significantly high preoperative LVEF (57.8 ± 0.8% vs. 55.8 ± 0.6%, *p* = 0.04) and elevated on-pump surgery rates (16.7% vs. 8.1%, *p* = 0.049). All other baseline variables, including age, sex, BMI, and comorbidities, did not exhibit significant differences between the groups (*p* > 0.05) (Table 3).

#### 3.1.4 High QFR at lesion site 1 vs. low QFR at lesion site 2

Significant differences were observed in 3-vessel disease prevalence (60.0% vs. 74.5%, *p* = 0.038) and hospital stay duration (26.2 ± 1.0 vs. 30.5 ± 1.5 days, *p* = 0.021). Other clinical parameters, including hypertension and prior myocardial infarction (MI), showed no statistical differences (*p* > 0.05) (Table 4).

#### 3.1.5 High QFR vs. low QFR at lesion site 2

Compared to patients with low QFR at Site 2, those with high QFR exhibited lower diabetes prevalence (21.3% vs. 42.5%, *p* = 0.009) and reduced 3-vessel disease (36.1% vs. 74.5%, *p* < 0.0001). Additionally, high QFR patients exhibited higher rates of concomitant surgery (50.8% vs. 17.9%, *p* < 0.0001), valvular surgery (41.0% vs. 14.2%, *p* = 0.0002), and on-pump CABG (47.5% vs. 13.2%, *p* < 0.0001) (Table 5).

Baseline characteristics for the remaining groups are presented in the Supplementary Material.

### 3.2 Clinical outcomes

During 5 years of follow-up, 107 MACCE events (cumulative incidence 22.2%) occurred among 481 patients, comprising 26 all-cause deaths (5.4%), 18 cardiovascular deaths (3.7%), 4 myocardial infarctions (0.8%), 22 strokes (4.6%), 5 repeat revascularizations (1.0%), and 28 hospitalizations for angina pectoris (5.8%). Stratification revealed 71 events in proximal LAD lesions (Site 1; *N* = 314) vs. 36 in mid-to-distal LAD lesions (Site 2; *N* = 167). Functional assessment showed 66 events in the low QFR group (QFR < 0.80; *n* = 337) compared to 41 in the high QFR group (QFR ≥ 0.80; *n* = 144). The integrated subgroups demonstrated: Subgroup A (High QFR at Lesion Site 1; *n* = 83) had 26 events, Subgroup B (Low QFR at Lesion Site 1; *n* = 231) had 45 events, Subgroup D (High QFR at Lesion Site 2; *n* = 61) had 15 events, and Subgroup C (Low QFR at Lesion Site 2; *n* = 106) had 21 events.

#### 3.2.1 High QFR vs. low QFR

**Figure 2:**
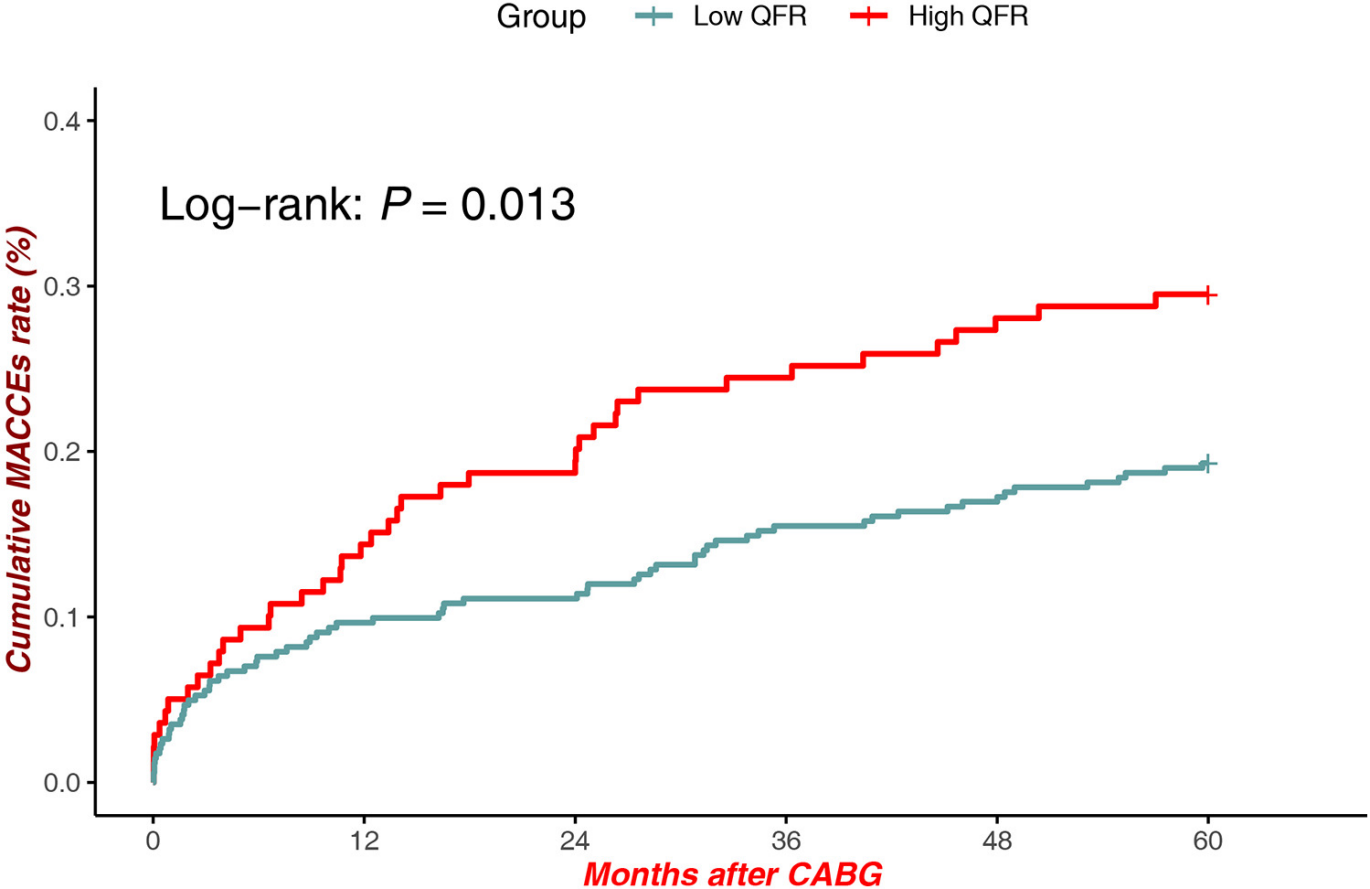
MACCEs in low QFR (all sites) vs. high QFR (All Sites).

Patients with high QFR exhibited significantly lower cumulative MACCEs compared to low QFR patients [Log-rank *P* = 0.013, HR = 1.053, 95% CI (0.705–1.572)]. The divergence in event rates emerged as early as 12 months post-CABG and continued to widen over the 60-month follow-up period (Figure 2).

#### 3.2.2 Lesion site 1 vs. lesion site 2

**Figure 3:**
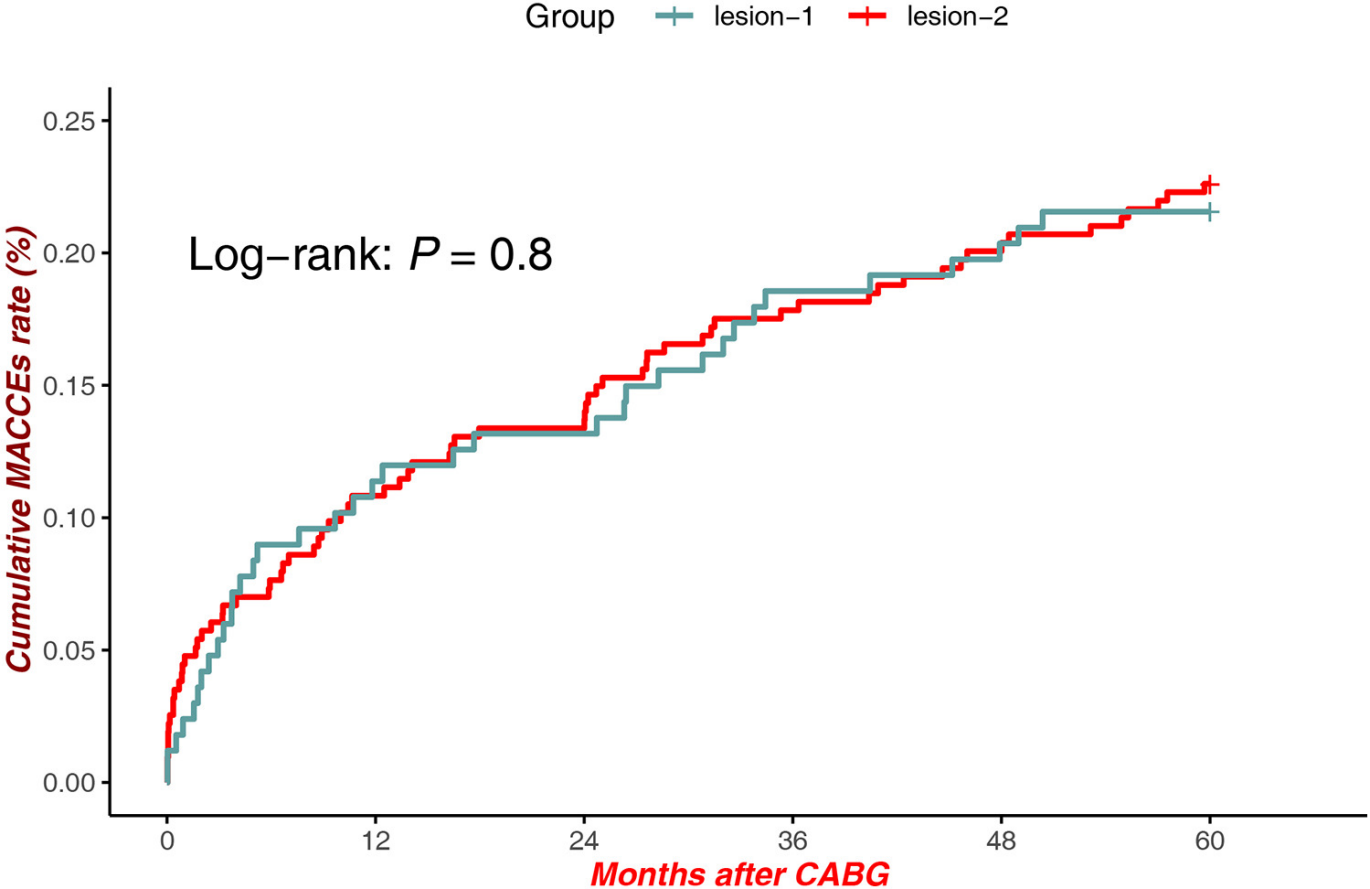
MACCEs in lesions (proximal LAD) vs. lesions (Mid-Distal LAD).

There were no statistically significant differences in MACCEs between patients with lesions at Site 1 and Site 2 [Log-rank *P* = 0.8, HR = 1.634, 95% CI (1.106–2.413)], suggesting comparable long-term clinical outcomes regardless of anatomical lesion location (Figure 3).

#### 3.2.3 High QFR vs. low QFR at lesion site 1

**Figure 4:**
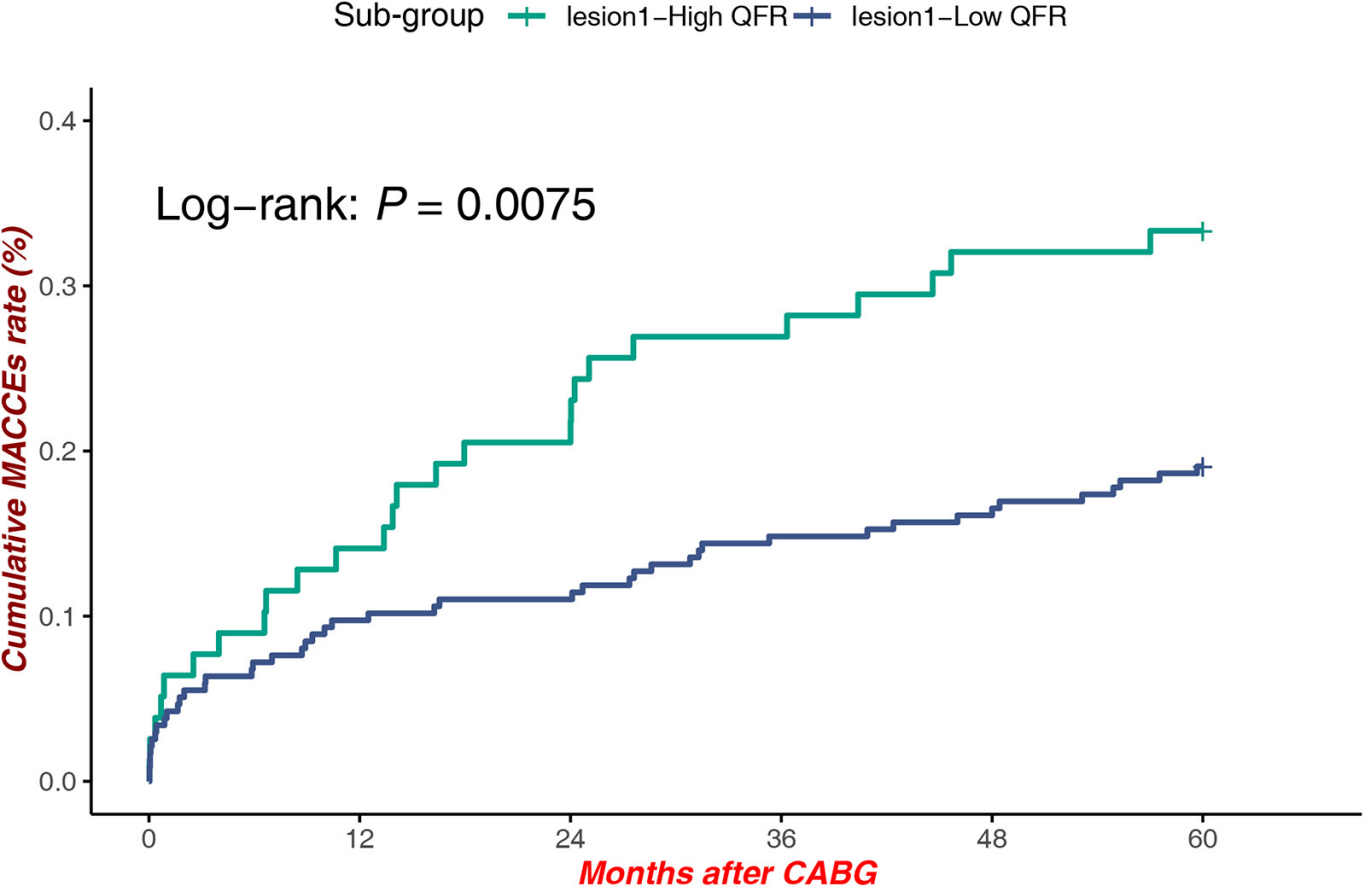
MACCEs in low QFR (proximal LAD) vs. high QFR (Proximal LAD).

Within lesions at Site 1, patients with high QFR demonstrated significantly reduced MACCEs compared to those with low QFR [Log-rank *P* = 0.0075, HR = 1.912, 95% CI (1.179–3.099)], underscoring the prognostic value of QFR stratification in this subgroup (Figure 4).

#### 3.2.4 High QFR at lesion site 1 vs. low QFR at lesion site 2

**Figure 5:**
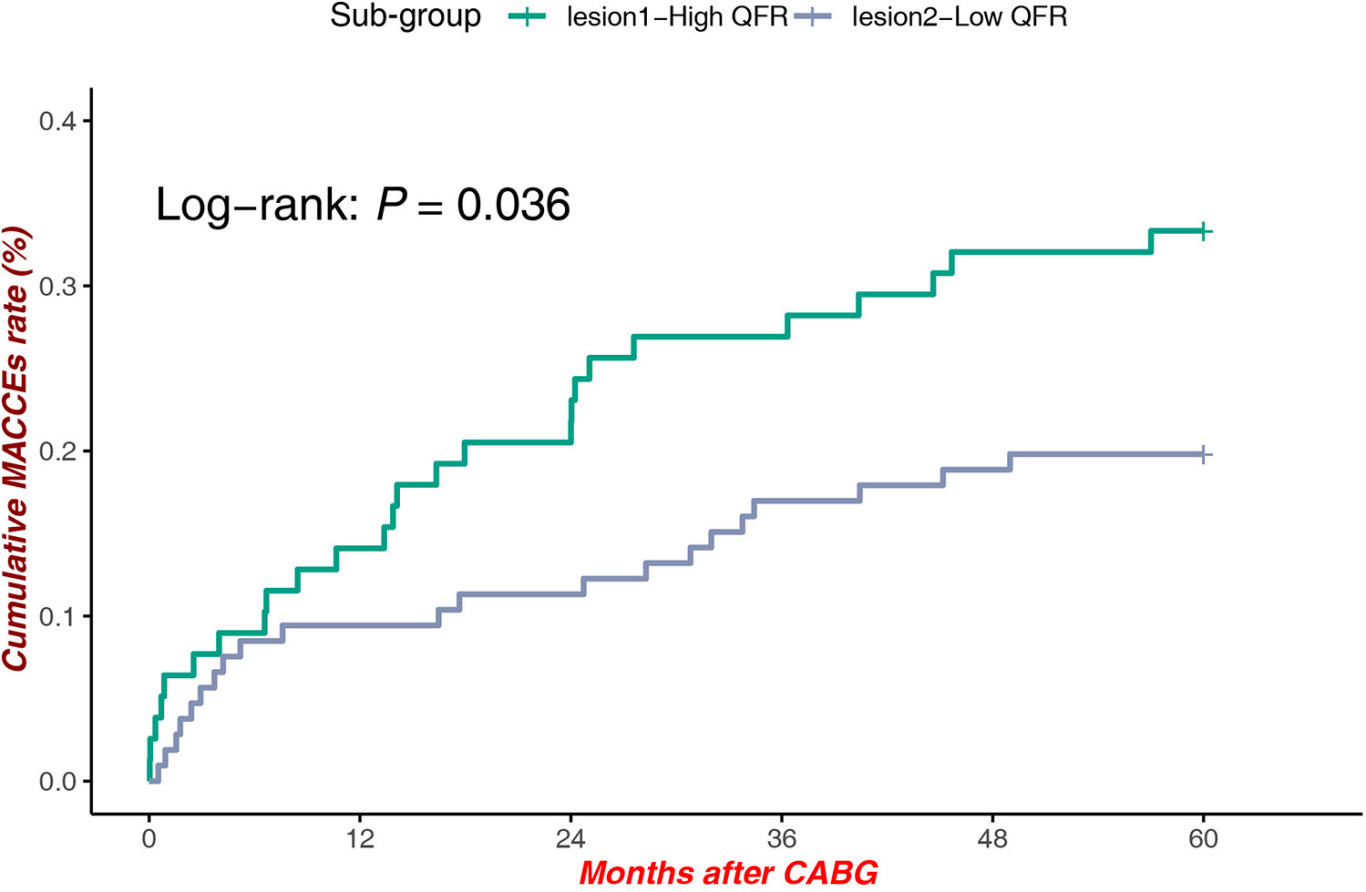
MACCEs in high QFR (proximal LAD) vs. low QFR (Mid-Distal LAD).

A significant survival benefit was observed in patients with high QFR at Site 1 compared to those with low QFR at Site 2 Low QFR patients [Log-rank *P* = 0.036, HR = 1.836, 95% CI (1.033–3.264)], highlighting the interplay between lesion location and hemodynamic significance in influencing clinical outcomes (Figure 5).

#### 3.2.5 High QFR vs. low QFR at lesion site 2

**Figure 6:**
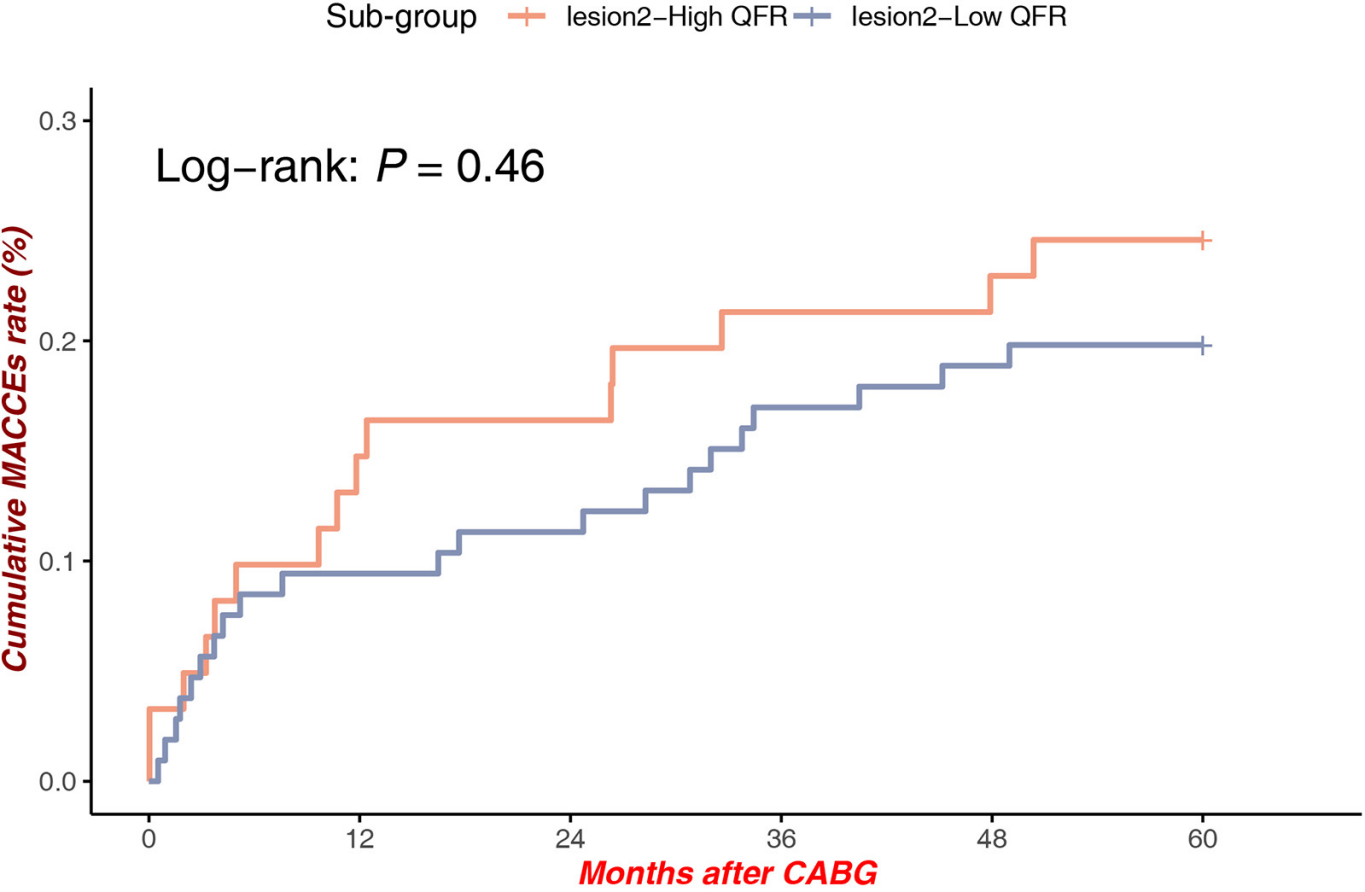
MACCEs in low QFR (Mid-distal LAD) vs. high QFR (Mid-Distal LAD).

Among patients with lesions at Site 2, QFR stratification was not associated with a statistically significantly difference in MACCEs rates [Log-rank *P* = 0.46, HR = 1.286, 95% CI (0.663–2.494)], indicating that anatomical and hemodynamic factors at this location may exert less impact on long-term outcomes (Figure 6).

Clinical outcomes for the remaining groups and multivariate cox regression for MACCEs with each individual event are presented in the Supplementary Material.

## 4 Discussion

In the current study, we found that LIMA grafting to quantitative flow ratio (QFR)-assessed functionally non-significant left anterior descending (LAD) arteries correlated with poor clinical outcomes compared with LIMA grafting to functionally significant LAD arteries. Notably, the anatomical location of coronary lesions did not significantly affect the long-term patient prognosis.

The principal objective of surgical coronary revascularization is to restore perfusion to the ischemic myocardial territories by deploying a low-resistance conduit in parallel alignment with the diseased coronary segment. Ideally, these adjunctive conduits must possess sufficient hemodynamic conductance to accommodate high-flow demands while maintaining minimal pressure attenuation throughout the distal implantation site. Competitive flow typically occurs when the resistance of the graft closely matches that of the stenosed native coronary artery (12). When analyzing the entire LAD using computational software, the functional significance of moderate stenosis localized to the mid-to-distal segment may be underestimated. This discrepancy arises from physiological pressure attenuation along the LAD. Which occurs naturally from its ostium to the distal segments under normal hemodynamics conditions (13). However, in QFR computation, the proximal pressure reference is typically derived from the vessel segment's origin, resulting in an overestimated baseline pressure compared to the actual pre-lesional pressure.

In this study, the analysis of QFR values across the entire LAD revealed that even when mid-to-distal segments were free of angiographically apparent proximal disease, the calculated QFR may be artifactually reduced. This phenomenon is attributed to coronary artery disease (CAD)-induced pathophysiological alterations (14), such as diffuse atherosclerosis or microvascular dysfunction (15), which may not manifest angiographically but contribute to distal pressure attenuation. Consequently, moderate stenosis in these segments may still induce myocardial ischemia due to impaired coronary flow reserve, potentially leading to angina pectoris despite the absence of hemodynamically significant proximal lesions.

Also, the lack of prognostic discrimination by QFR in mid-to-distal lesions may arises from: computational limitations in modeling serial stenoses. For improved assessment, we advocate combining invasive microcirculatory evaluation (IMR) with intravascular imaging (IVUS/OCT)-guided functional analysis to enable personalized ischemia risk stratification. Even when QFR values exceed the threshold 0.8, experienced cardiac surgeons may opt for LIMA grafting to the LAD following a multidisciplinary evaluation that integrates clinical manifestations, hemodynamic profiles, and ancillary diagnostic findings. This decision underscores the importance of individualized clinical judgment, which prioritizes anatomical complexity, ischemia burden, and long-term prognostic outcomes over isolated QFR thresholds alone in selected cases in select patients. Based on our findings, preoperative QFR assessment is recommended when feasible to optimize revascularization strategy selection and improve prognostic outcomes, without adding procedural risk. While QFR incurs higher costs, its integration should be guided by multidisciplinary evaluation and shared decision-making with patients, considering individual economic circumstances.

Conventionally, ostial and proximal coronary lesions are presumed to induce larger ischemic territories compared to mid-to-distal lesions, resulting in more severe clinical manifestations and poorer prognostic outcomes (16). However, the present study did not reveal significant differences in clinical outcomes between these lesion subtypes. This finding may be attributed to the effective surgical resolution of myocardial ischemia through comprehensive revascularization, as well as the high long-term graft patency rates associated with LIMA-LAD anastomosis (17). These factors collectively mitigate the impact of lesion location on ischemia-driven adverse events.

## 5 Limitation

This study has several limitations that warrant acknowledgment. First, its retrospective and observational design introduces inherent risks of selection bias, confounding by indication, and potential underreporting of adverse events, particularly given the limited sample size within certain subgroups. Second, the accuracy of QFR measurements is dependent on angiographic image quality and acquisition protocols, which could not be standardized in this retrospective analysis. Third, the number of clinical events was insufficient for robust multivariable adjustment of baseline differences between QFR groups (Supplementary Table S6), resulting in underpowered Cox regression analyses and residual confounding. Fourth, the follow-up duration (median 5 years) restricts the evaluation of long-term graft patency and late-term MACCEs dynamics, necessitating validation through longitudinal studies. Fifth, unmeasured confounders—such as microvascular resistance (18–20), plaque vulnerability, or genetic predispositions—were not adjusted for, potentially influencing QFR-outcome associations. Additionally, the single-center cohort limits the generalizability of these findings, underscoring the need for multicenter validation studies employing standardized angiographic acquisition protocols and adjudicated clinical endpoint reporting to enhance reproducibility and generalizability.

## 6 Conclusions

This study demonstrates that patients undergoing LIMA to LAD grafting with a preoperative LAD QFR > 0.80 exhibited poorer long-term prognoses, consistent with prior observational studies' evidence. Notably, in patients with mid-to-distal LAD lesions, QFR values exceeding 0.80 showed no significant association with adverse clinical outcomes. Furthermore, lesion location (proximal vs. mid-to-distal) did not independently influence long-term prognosis in the LIMA-LAD cohort.

## Data Availability

The raw data supporting the conclusions of this article will be made available by the authors, without undue reservation.
