# Supplementary material for: Impact of left anterior descending lesion location on midterm outcomes in patients undergoing left internal mammary artery grafting: a five-year cohort study integrating quantitative flow ratio assessment
Source: Front Cardiovasc Med. 2025 Aug 11;12:1605573. doi: 10.3389/fcvm.2025.1605573 (PMC12375600; doi:10.3389/fcvm.2025.1605573)
Supplement: Supplementary file 1 [file Datasheet1.pdf]

Supplementary Material

1 Supplementary Figures and Tables

1.1 Supplementary Figures

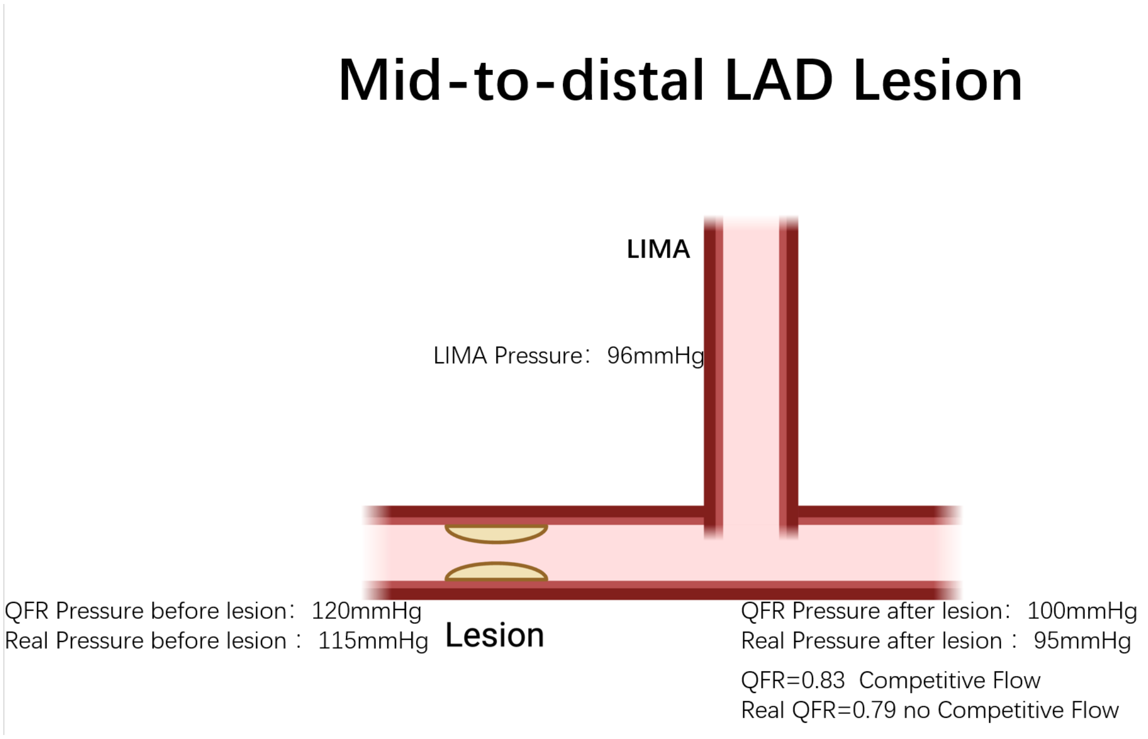

Supplementary Figure 1. Mechanism of competitive blood flow at the site of mid- and distal lad lesions.

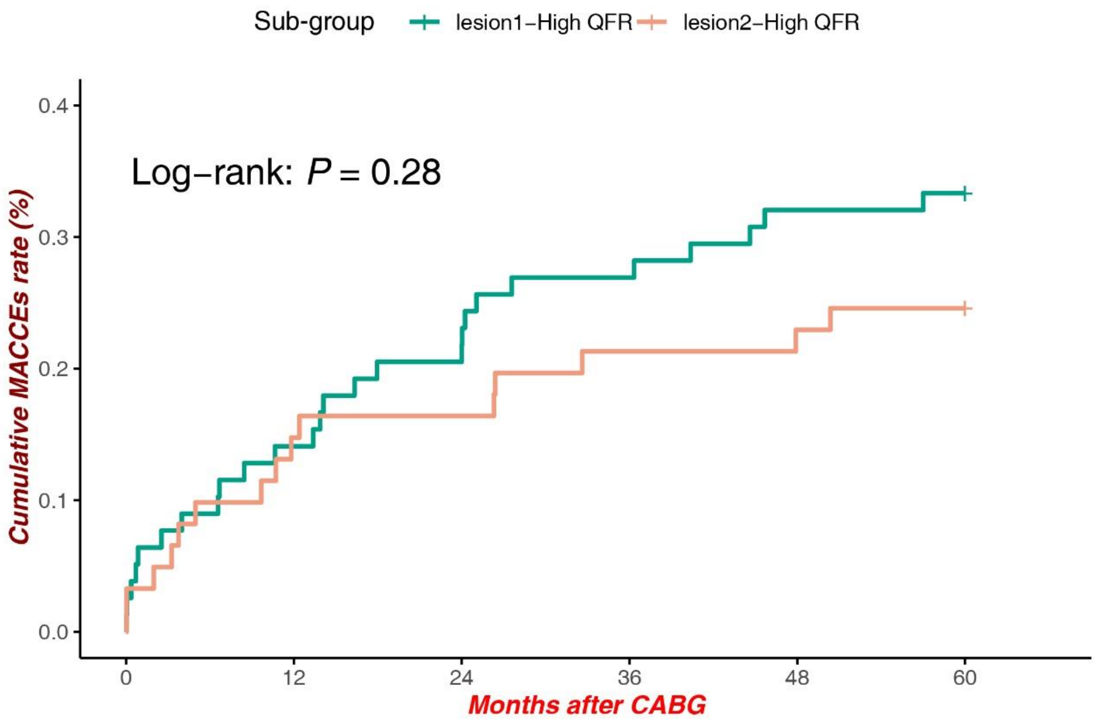

Supplementary Figure 2. MACCEs in High QFR (Proximal LAD) vs. High QFR (Mid-Distal LAD).

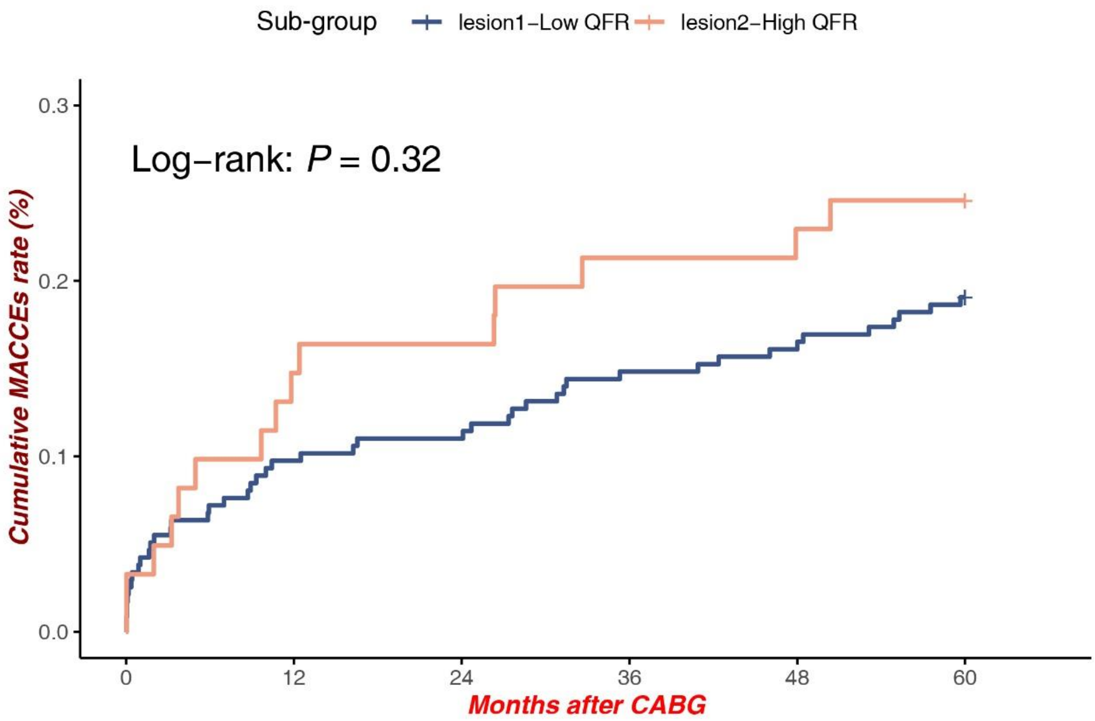

Supplementary Figure 3. MACCEs in Low QFR (Proximal LAD) vs. High QFR (Mid-Distal LAD).

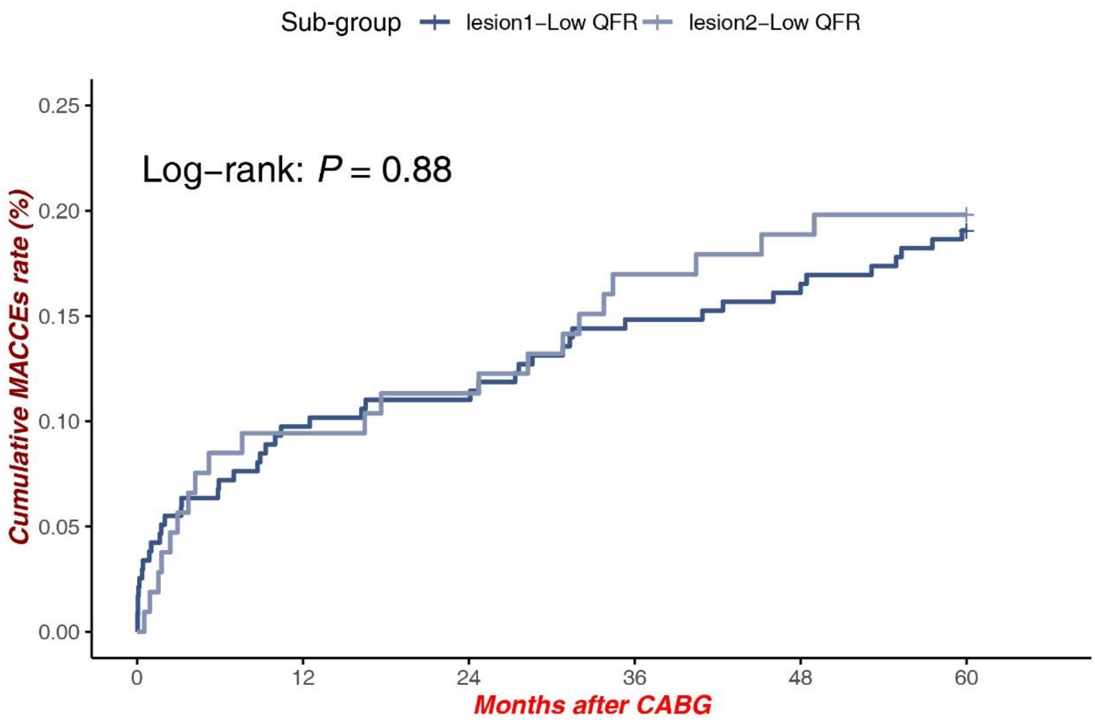

Supplementary Figure 4. MACCEs in Low QFR (Proximal LAD) vs. Low QFR (Mid-Distal LAD).

1.2 Supplementary Tables

Supplementary Table 1. Multivariate cox regression for MACCEs with each individual event comparing Lesion Sites.

	Lesion Site 1 (n=134)	Lesion Site 2 (n=167)	HR(95%CI) for QFR>0.8	P value
MACCEs	65(20.7%)	32(19.2%)	1.13 (0.75–1.69)	0.56
All cause death	19(6.1%)	7(4.2%)	1.49 (0.69–3.21)	0.31
Cardiovascular death	11(3.5%)	7(4.2%)	0.95 (0.37–2.44)	0.92
Myocardial infarction	3(0.9%)	1(0.6%)	1.35 (0.14–12.97)	0.8
Stroke	13(4.1%)	9(5.4%)	0.65 (0.29–1.43)	0.28
Repeat revascularization	5(1.6%)	0(0%)		
Rehospitalization for Angina Pectoris	22(7.0%)	6(3.6%)	2.35 (0.95–5.82)	0.064

MACCEs, Major Adverse Cardiac and Cerebrovascular Events; HR, Hazard Ratio; CI, Confidence Interval; LVEF, Left Ventricular Ejection Fraction.

Supplementary Table 2. Multivariate cox regression for MACCEs with each individual event comparing QFR.

	High QFR (n=144)	LOWQFR (n=337)	HR(95%CI) for QFR>0.8	P value
MACCEs	36(25.9%)	61(17.8%)	1.66 (1.12–2.46)	0.012
All cause death	9(6.5%)	17(5.0%)	1.08 (0.52–2.28)	0.83
Cardiovascular death	7(5.0%)	11(3.2%)	1.45 (0.57–3.72)	0.44
Myocardial infarction	0(0%)	4(1.2%)		
Stroke	5(3.6%)	17(5.0%)	0.72 (0.29–1.83)	0.5
Repeat revascularization	0(0%)	5(1.5%)		
Rehospitalization for Angina Pectoris	13(9.4%)	15(4.4%)	2.28 (1.09–4.77)	0.029

MACCEs, Major Adverse Cardiac and Cerebrovascular Events; HR, Hazard Ratio; CI, Confidence Interval; LVEF, Left Ventricular Ejection Fraction.

Supplementary Table 3. Comparison of Baseline Characteristics between lesion Site 1(Proximal LAD) and lesion Site 2 (Mid-to-Distal LAD) in High QFR.

	lesion Site 1-High QFR (n=83)	lesion Site 2-High QFR (n=61)	p value
Age (years)	63.1±0.8	64.8±0.9	0.162
Male	62(79.5%)	44(72.1%)	0.417
BMI (kg/m²)	25.5±0.3	25.3±0.4	0.765
Hypertension	47(60.3%)	31(50.8%)	0.347
Hypercholesterolemia	2(2.6%)	34(55.7%)	0.807
Diabetes mellitus	26(33.3)	13(21.3%)	0.169
Prior MI	8(10.3%)	4(6.6%)	0.641
Smoking	23(29.5%)	15(24.6%)	0.652
Cerebrovascular diseases	12(15.4%)	5(8.2%)	0.306
Prior left coronary artery PCI	7(9.0%)	4(6.6%)	0.836
Preoperative LVEF,%	57.8±0.8	57.0±0.9	0.505
Concomitant surgery	18(23.1%)	31(50.8%)	0.001
Valvular surgery	12(15.4%)	25(41.0%)	0.001
Aortic surgery	0(0%)	1(1.6%)	
Others	4(5.1%)	2(3.3%)	0.911
On-pump	13(16.7%)	29(47.5%)	<0.001
3-vessel disease	46(60.0%)	22(36.1%)	0.012
Hospital complications	39(50.0%)	37(60.1%)	0.280
Hospital stays	26.2±1.0	29.3±1.8	0.118
NYHA class			0.442
I	2(2.6%)	1(1.6%)	
II	61(78.2%)	41(67.2%)	
III	12(15.4%)	15(24.6%)	
IV	3(3.8%)	4(6.6%)	

MACCEs, Major Adverse Cardiac and Cerebrovascular Events; HR, Hazard Ratio; CI, Confidence Interval; LVEF, Left Ventricular Ejection Fraction.

Supplementary Table 4. Comparison of Baseline Characteristics between High QFR in Lesion Site 2 (Mid-to-Distal LAD) and Low QFR in Lesion Site 1 (Proximal LAD).

	lesion Site 2-High QFR (n=61)	lesion Site 1-Low QFR (n=231)	p value
Age (years)	64.8±0.9	63.1±0.5	0.104
Male	44(72.1%)	178(75.4%)	0.717
BMI (kg/m²)	25.3±0.4	25.6±0.2	0.600
Hypertension	31(50.8%)	156(66.1%)	0.040
Hypercholesterolemia	34(55.7%)	6(2.5%)	>0.999
Diabetes mellitus	13(21.3%)	94(39.8%)	0.011
Prior MI	4(6.6%)	32(13.6%)	0.202
Smoking	15(24.6%)	93(39.4%)	0.046
Cerebrovascular diseases	5(8.2%)	31(13.3%)	0.405
Prior left coronary artery PCI	4(6.6%)	11(4.7%)	0.783
Preoperative LVEF,%	57.0±0.9	55.8±0.6	0.249
Concomitant surgery	31(50.8%)	32(13.6%)	<0.001
Valvular surgery	25(41.0%)	21(8.9%)	<0.001
Aortic surgery	1(1.6%)	1(0.4%)	0.875
Others	2(3.3%)	4(1.7%)	0.784
On-pump	29(47.5%)	19(8.1%)	<0.001
3-vessel disease	22(36.1%)	176(74.6%)	<0.001
Hospital complications	37(60.1%)	122(51.7%)	0.268
Hospital stays	29.3±1.8	29.1±0.8	0.895
NYHA class			0.697
I	1(1.6%)	4(1.7%)	
II	41(67.2%)	176(74.6%)	
III	15(24.6%)	45(19.1%)	
IV	4(6.6%)	11(4.7%)	

MACCEs, Major Adverse Cardiac and Cerebrovascular Events; HR, Hazard Ratio; CI, Confidence Interval; LVEF, Left Ventricular Ejection Fraction.

Supplementary Table 5. Comparison of Baseline Characteristics between lesion Site 1(Proximal LAD) and lesion Site 2 (Mid-to-Distal LAD) in Low QFR.

	lesion Site 1-Low QFR (n=231)	lesion Site 2-Low QFR (n=106)	p value
Age (years)	63.1±0.5	62.7±0.8	0.778
Male	178(75.4%)	76(71.7%)	0.552
BMI (kg/m²)	25.6±0.2	28.1±2.9	0.361
Hypertension	156(66.1%)	66(62.3%)	0.572
Hypercholesterolemia	6(2.5%)	4(3.8%)	0.781
Diabetes mellitus	94(39.8%)	45(42.5%)	0.736
Prior MI	32(13.6%)	18(17.0%)	0.507
Smoking	93(39.4%)	39(36.8%)	0.734
Cerebrovascular diseases	31(13.3%)	16(15.1%)	0.751
Prior left coronary artery PCI	11(4.7%)	4(3.8%)	0.932
Preoperative LVEF,%	55.8±0.6	55.7±0.8	0.097
Concomitant surgery	32(13.6%)	19(17.9%)	0.377
Valvular surgery	21(8.9%)	15(14.2%)	0.203
Aortic surgery	1(0.4%)	0(0%)	
Others	4(1.7%)	2(1.9%)	>0.999
On-pump	19(8.1%)	14(13.2%)	0.195
3-vessel disease	176(74.6%)	79(74.5%)	>0.999
Hospital complications	122(51.7%)	64(60.4%)	0.170
Hospital stays	29.1±0.8	30.5±1.5	0.021
NYHA class			0.431
I	4(1.7%)	2(1.9%)	
II	176(74.6%)	70(66.0%)	
III	45(19.1%)	28(26.4%)	
IV	11(4.7%)	6(5.7%)	

MACCEs, Major Adverse Cardiac and Cerebrovascular Events; HR, Hazard Ratio; CI, Confidence Interval; LVEF, Left Ventricular Ejection Fraction.

Supplementary Table 6. Initial Comprehensive and Final Parsimonious Cox Models for MACE Risk in High- versus Low-QFR Groups: Analysis of Combined Lesion Sites.

Model/Analysis	Covariate	HR	95% CI	p-value	Schoenfeld p
**Initial Cox Model (481 observations, 107 events)**	Hospital stays	1.024	1.01–1.04	<0.005	0.75
	Age	1.015	0.99–1.04	0.254	0.05
	BMI	0.999	0.98–1.02	0.942	0.79
	Preoperative LVEF	0.988	0.97–1.01	0.309	0.04
	On pump	1.004	0.45–2.26	0.992	0.34
	Concomitant surgery_	0.438	0.14–1.34	0.149	0.63
	Valvular surgery	2.157	0.68–6.80	0.190	0.83
	Aortic surgery	NA	NA	0.996	1.00
	Others_	2.367	0.62–9.01	0.206	0.55
	3 vessel disease	1.140	0.71–1.82	0.585	<0.005
	Hospital complications_	1.109	0.74–1.65	0.612	0.52
	Prior left coronary artery PCI	2.364	1.21–4.61	0.012	0.17
	Men	0.885	0.55–1.43	0.618	0.56
	Hypertension	1.082	0.72–1.64	0.708	0.10
	Hypercholesterolemia	1.554	0.56–4.29	0.395	0.19
	Diabetes mellitus	1.051	0.69–1.60	0.817	0.09
	Cerebrovascular diseases 1	0.522	0.26–1.04	0.063	0.81
	Smoking	0.910	0.57–1.45	0.690	0.25
	Prior MI	0.732	0.39–1.39	0.339	0.63
	NYHA class	0.712	0.49–1.04	0.077	0.57
**Refined Cox Model (481 observations, 107 events)**	Prior left coronary artery PCI	2.223	1.18–4.19	0.013	0.24
	Men	0.857	0.56–1.31	0.477	0.75
	Hospital stays	1.022	1.01–1.03	<0.005	0.96
	Cerebrovascular diseases	0.540	0.27–1.06	0.074	0.56
	Age	1.015	0.99–1.04	0.262	0.03
	NYHA class	0.718	0.50–1.04	0.078	0.84
**Log-rank Test**					
	QFR groups	-	-	0.011	-

HR = hazard ratio; CI = confidence interval. Schoenfeld p-values assess the proportional hazards assumption ($p > 0.05$ indicates assumption met). The refined model includes covariates with $p < 0.10$

or clinical relevance from the initial model. The Log-rank test ($p = 0.011$) confirms significant differences in survival curves between QFR > 0.8 and QFR ≤ 0.8 groups.

Covariates were selected based on statistical significance ($p < 0.10$) in the initial Cox model and clinical relevance. group, Hospital stays, and Prior left coronary artery PCI ($p < 0.05$) and Cerebrovascular diseases and NYHA class ($p < 0.10$) were retained as significant predictors. Age and Men were included due to their clinical importance in cardiovascular outcomes, despite $p > 0.10$. Covariates with $p > 0.10$ and no strong clinical rationale (e.g., BMI, Aortic surgery, Preoperative LVEF) were excluded to avoid overfitting, as shown in Supplementary Table 6.
